# Endovascular repair of de novo post-stenotic aortic coarctation aneurysms with complex collateral supply: two cases with long and medium term follow-up

**DOI:** 10.1186/s42155-020-00193-4

**Published:** 2021-01-11

**Authors:** Omar Abdel-Hadi, John Thomson, Simon J. McPherson

**Affiliations:** 1 Department of Radiology, Leeds Teaching Hospitals NHS Trust, Leeds General Infirmary, Great George Street, Leeds, West Yorkshire LS1 3EX UK; 2 Department of Adult Congenital Heart Disease, Leeds Teaching Hospitals NHS Trust, Leeds General Infirmary, Leeds LS1 3EX, West Yorkshire. LS1 3EX UK; 3 University of Leeds, Leeds, UK

**Keywords:** Percutaneous, Thoracic coarctation aneurysm, Thoracic aortic stent graft/repair, TEVAR, Aortic coarctation stent, Embolisation

## Abstract

**Purpose:**

To report the technical details and outcomes of the endovascular repair of two cases of de novo post-stenotic aortic coarctation aneurysms complicated by complex collateral supply.

**Case presentations:**

Two patients with thoracic aortic aneurysms complicated by complex aneurysm sac collaterals distal to a previously untreated thoracic aortic coarctation have been treated at our institution. Open surgical intervention was deemed to carry a high risk of haemorrhage due to the degree and complexity of arterial collateralisation. In the first case, selective embolisation of collateral vasculature was performed prior to successful exclusion of the aneurysm with a thoracic endovascular stent-graft and then balloon-expandable stent dilatation of the coarctation stenosis. In the second case, the additional technique of using a jailed sheath within the aneurysm sac allowed for selective embolisation of previously inconspicuous collaterals after deployment of the stent-graft and stent combination.

**Results:**

Technical success was achieved in both patients with successful occlusion of the aneurysm, with no recorded complications or aneurysm sac perfusion in the long and medium term follow up periods respectively.

**Conclusion:**

De novo post stenotic aortic coarctation aneurysms are rare. Endovascular repair is a safe and durable technique that provides a less invasive alternative to open surgical repair. The use of a jailed sheath allows for complete selective embolisation of complex collaterals avoiding a type II aneurysm endoleak.

## Background

Thoracic aortic coarctation (TAC) is a focal aortic constriction typically at the ductus or ligamentum arteriosum. It accounts for 5–8% of congenital cardiac defects (Tynan et al. 1990; Warnes et al. 2008). Consequent increased cardiac afterload results in hypertension, left ventricular hypertrophy, and congestive cardiac failure (Warnes et al. 2008). Most present in childhood when open surgical repair is often performed. Surgical approaches include excision with end-to-end anastomosis, extended end to side anastomosis (particularly in infants), synthetic patch aortoplasty, subclavian artery flap aortoplasty and interposition tube grafts (Khavandi et al. 2013). Presentation as an adult occurs in 15–20% who are asymptomatic in childhood. The natural history of untreated adult TAC is a mortality rate approaching 75% by the fifth decade (Campbell 1970; Erbel et al. 2014)., The preferred treatment in older children and adult patients is endovascular due to its lower morbidity and mortality compared with surgery (Forbes and Gowda 2014).

Post-surgical aneurysms in TAC are well recognised but aneurysms are much rarer in untreated cases (Khavandi et al. 2013; Turner and Gaines 2007). Thoracic Endovascular Aneurysm Repair (TEVAR) is the accepted first-line treatment for post-surgical aneurysms, given the mortality rate of 14% for repeat open surgery (Ince et al. 2003). Successful open surgery for de-novo TAC associated aneurysms is limited to case reports (Ruys et al. 2014; Ananiadou et al. 2012; Careaga-Reyna et al. 2009). Stent-grafts in combination with other endovascular techniques offer a flexible alternative to cope with anatomic complexity (Kutty et al. 2008).

We report two patients with de novo aneurysms distal to a TAC, with associated extensive aneurysm collateralisation, treated by complex endovascular repair.

## Case presentation

### Case 1

**Fig. 1:**
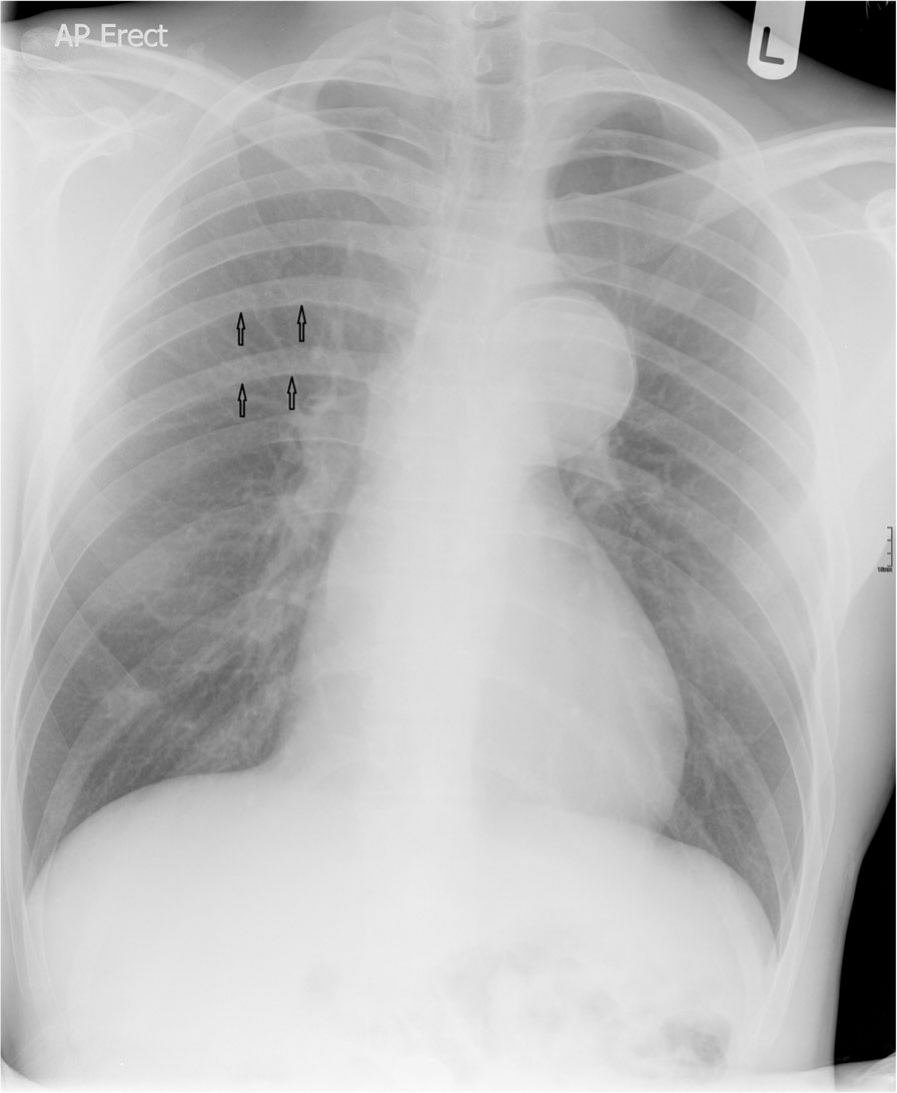
Presenting chest radiograph with a large calcified aortic aneurysm and rib notching in the right upper zone (Black Arrows)

**Fig. 2:**
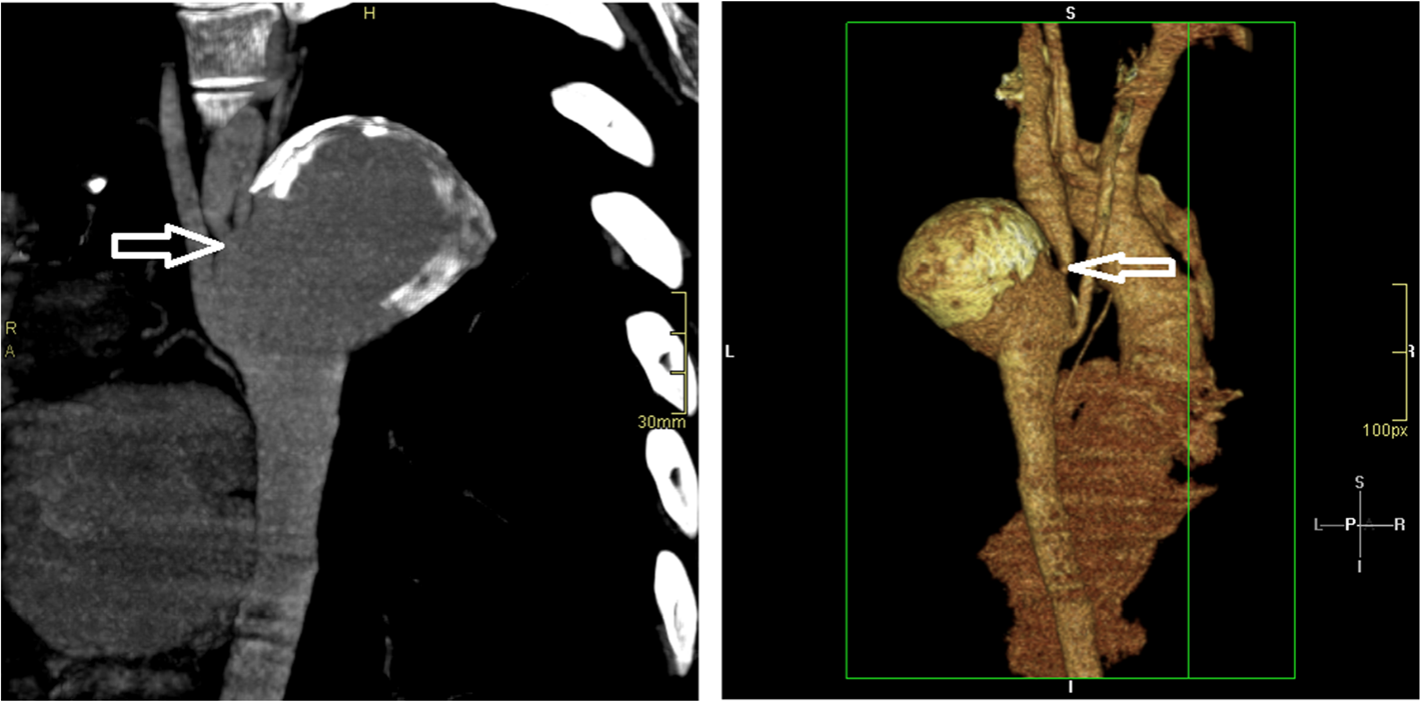
Cross sectional reformats demonstrating aneurysm formation in an aortic coarctation (White arrows)

A 28-year-old male presented in 2010 with chest pain following amphetamine use. CXR revealed a grossly enlarged and peripherally calcified descending thoracic aortic aneurysm with rib notching (Fig. 1). CT angiogram confirmed a 5.7 cm calcified aneurysm distal to a 5 mm lumen TAC (Fig. 2), and florid predominantly posterior collaterals. The chest pain persisted despite medical therapy. Alternative causes for the pain were excluded. Discussion at the complex cardiothoracic disease multi-disciplinary team (MDT) meeting concluded that the extent of collateralisation posed a high risk of uncontrollable bleeding. An endovascular approach was preferred, this was agreed with the patient.

Under general anaesthesia (GA), with a cardiothoracic team on stand-by, an 18Fr sheath (Cook, Bloomington, IN) was inserted via a right common femoral artery (CFA) surgical cut down with a left CFA 6Fr sheath. The procedure predates our routine use of large arterial access closure devices. The small TAC lumen was hard to identify within the large aneurysm. It was eventually crossed with a 4Fr vertebral catheter and hydrophilic wire (Terumo, Japan). A 260 cm Amplatz wire (Cook) was placed across the TAC into the left subclavian artery (LSCA) for emergency TEVAR or balloon control in the event of bleeding.

**Fig. 3:**
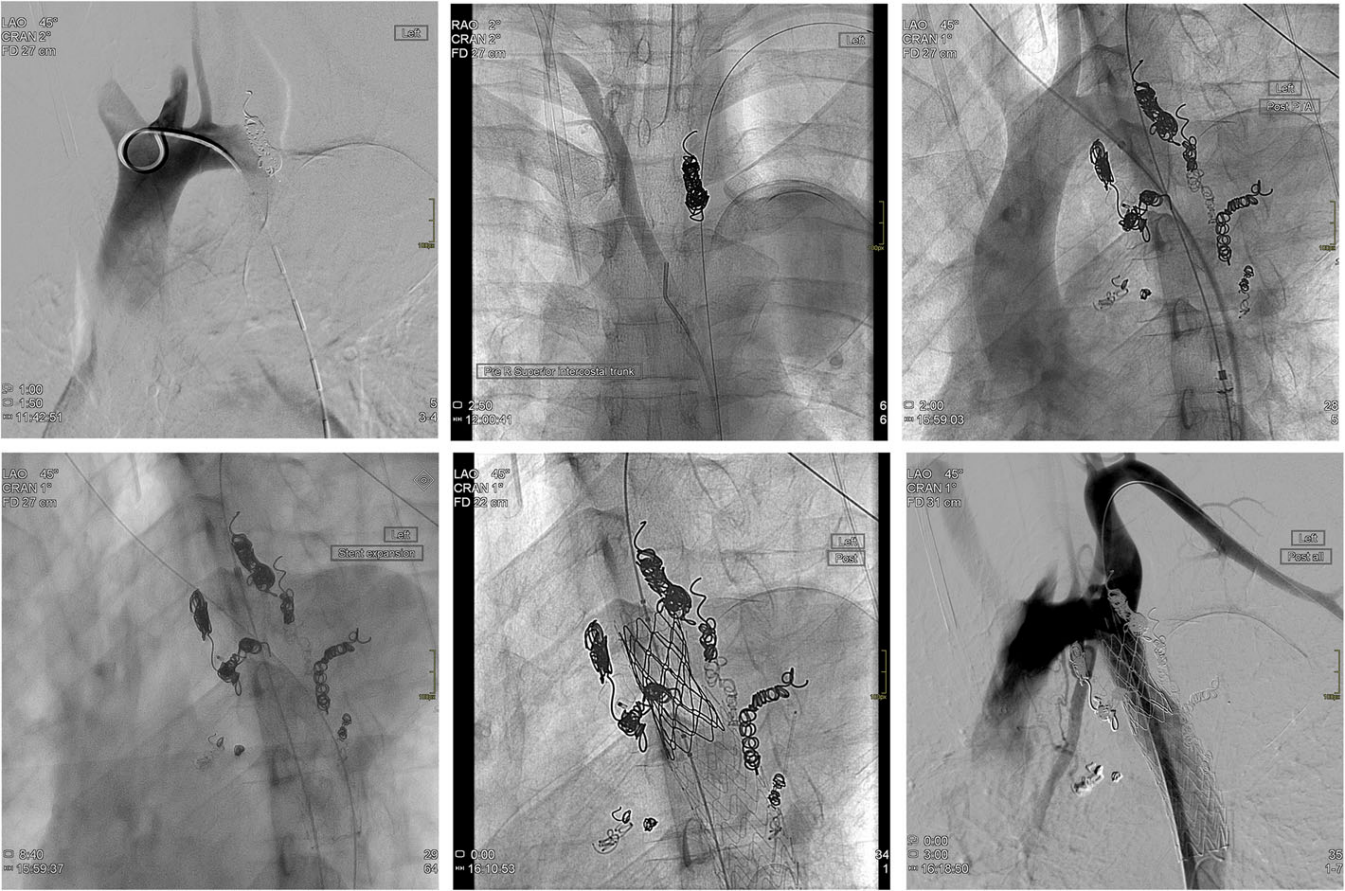
Sequential embolization of collaterals followed by insertion of self-expanding and then balloon mounted stent grafts. Successful embolization and stent-grafting with no endoleak

Over 10 intercostal and bronchial arteries arising from the aneurysm sac were coiled using a combination of 0.035 in. Nester (Cook), 0.035 MReye coils (Cook) Trufill platinum micro coils (Cordis, Miami, Florida) and one Amplatz AVP1 10 mm plug (AGA Medical, Plymouth, Minnesota,). TAC 8 mm pre-dilatation facilitated TEVAR delivery. A 21 mm × 100 mm Gore TAG device (W. L. Gore, Flagstaff, AZ) was deployed immediately distal to the LSCA. A 39 mm length uncovered CP stent (NuMED, Hopkinton, New York,) mounted onto a 16 mm outer BIB balloon (NuMED) was delivered flush with the cranial stent graft with serial inflations leading to good apposition resulting in almost complete abolition of the coarctation and exclusion of the aneurysm (Fig. 3). Procedure time was 5 h. CT angiography at 72 h confirmed complete aneurysm exclusion.

Over 7 years CT surveillance showed aneurysm sac shrinkage to 4.2 cm with no endoleak. Eight years post operatively the patient presented with left sided weakness secondary to emboli from culture negative infection associated with intravenous drug use. Echocardiography identified a mitral valve vegetation. CT angiogram and PET-CT showed no evidence of infection at the site of the coarctation and aneurysm repair. Treatment was with long term IV antibiotics. At 10 years CT follow-up the aneurysm remains excluded and at a stable reduced size with no recurrence of the TAC.

### Patient 2

**Fig. 4:**
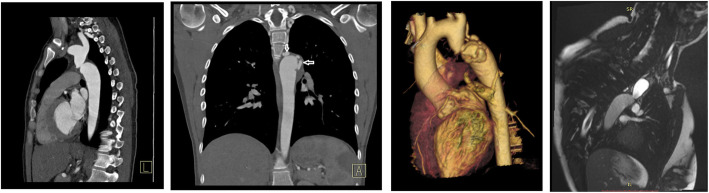
Pre operative imaging demonstrating a significant post ductal aortic coarctation. Multiple aneurysms surrounding the coarctation (white arrows)

A 29 year old female was referred from another regional specialist cardiothoracic centre. She was well until 2017 when she developed a painful left foot. Septic emboli due a Streptococcal sanguis endarteritis at the site of previously undiagnosed coarctation, with a 3.5 cm aneurysm distal to it, was diagnosed. The aneurysm had numerous large collateral vessels, and two lateral out-pouchings measuring 6 x19mm and 11 × 9 mm which were considered most likely to be pseudoaneurysms (Fig. 4). After 6 months of intravenous antibiotic therapy and imaging surveillance, she was referred for operative treatment. CT angiogram demonstrated an 8 mm coarctation 3 cm distal to the LSCA with an unchanged aneurysm (Fig. 4). Considering the infectious clinical presentation, preference to avoid an interposition graft with a suture line and high bleeding risk the MDT offered an endovascular repair.

**Fig. 5:**
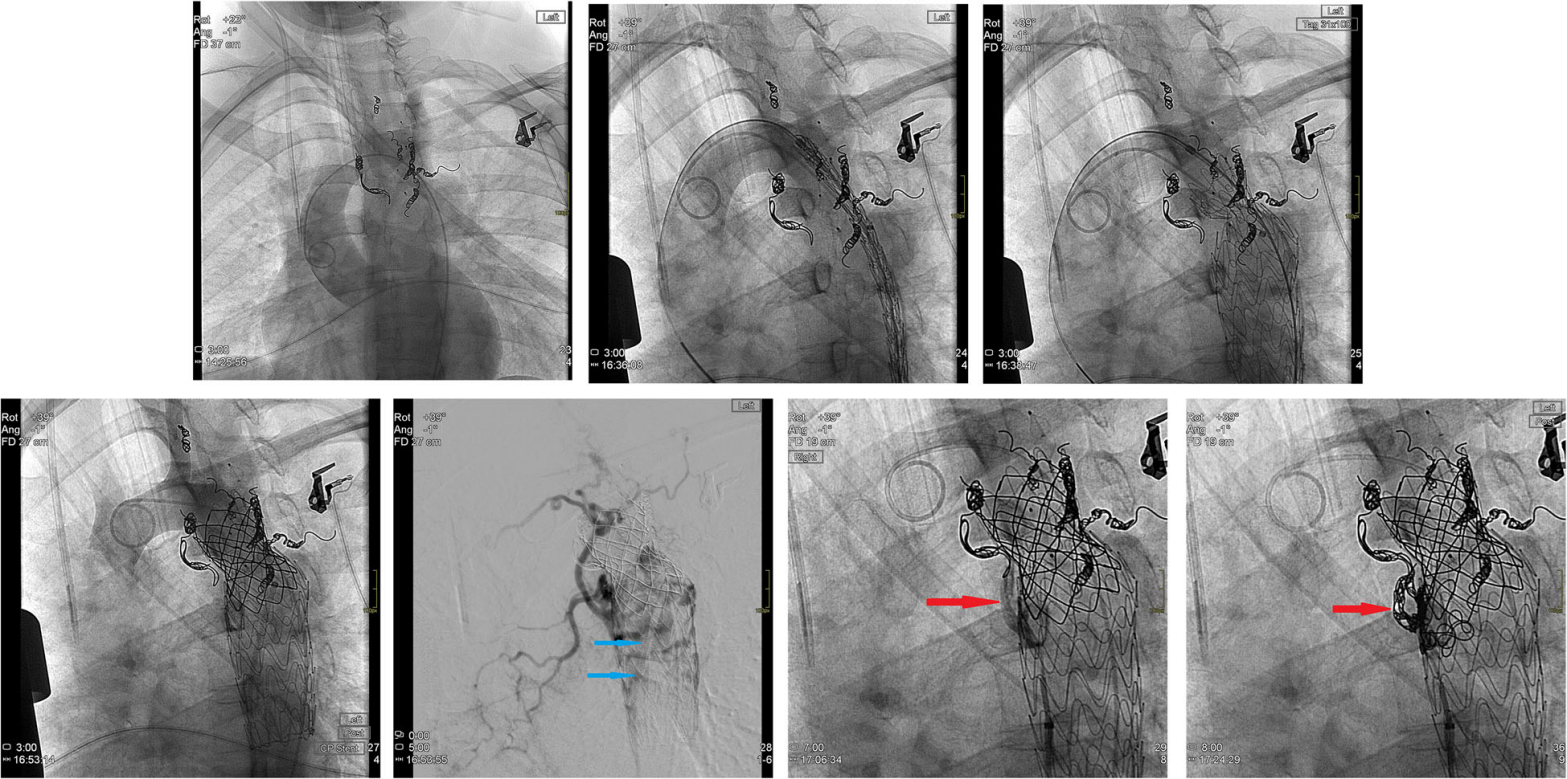
Image series demonstrating embolisation and stent graft insertion to successfully occlude the aneurysm and dilate the aortic coarctation. Red arrow. represents persisting collaterals pre and post embolisation, Blue arrow corresponds to the position of the jailed sheath

Under GA, aneurysm sac collaterals were embolised via a 5Fr right CFA sheath using Trufill micro-coils (Cordis) and Amplatzer AVP4 vascular plugs (AGA Medical). These included superior intercostal arteries, bronchial arteries, broncho-intercostal arteries, and posterior intercostals connecting to the internal mammary arteries. No residual aneurysm sac collaterals were seen on completion aortic angiography. Surgical formation of a 10 mm distal aortic conduit was required for TEVAR delivery as the 5 mm iliac arteries were too narrow in calibre. The tip of an 80 cm 5Fr Flexor sheath (Cook) from a left CFA access was left within the aneurysm sac in case collateral embolisation was incomplete. A 31 mm × 100 mm Gore TAG stent graft (W. L. Gore) was deployed distal to the origin of the LSCA. A 39 mm length covered CP stent (NuMED) was dilated to 24 mm to treat the aortic coarctation (Fig. 5).

After exclusion of the aneurysm, angiography via the long sheath in the aneurysm sac demonstrated two patent non-embolised collateral branches (Fig. 5). These were embolised using a catheter and Progreat microcatheter (Terumo, Japan) combination and 10 × 14 micronester coils (Cook). No complication occurred. A 5 day post-operative CT showed no endoleak. She remains asymptomatic over 3 years of follow-up with a stable aneurysm sac and no endoleak.

## Discussion

The two cases were performed 7 years apart reflecting the rarity of de novo TAC aneurysms with complex aneurysm sac collaterals. Endovascular intervention was a safe and durable treatment.

A balloon expandable stent graft was used in the second case as the seal zone was shorter than in the first case and if slippage of the TAG stent graft had occurred during the balloon expandable stent-graft deployment the seal zone would have been maintained.

In case one, successful embolisation of all complex collateral arteries was achieved prior to aneurysm and TAC treatment. In case two, persistent collaterals were present despite apparent complete embolisation before the aneurysm and TAC treatment. The aneurysm sac “jailed” sheath ensured two further branches were embolised post stent-grafting. Without a jailed sheath embolisation of the persisting collaterals would not have been possible with a high risk of type 2 endoleak. The jailed sheath allowed alternative embolisation techniques, such as cohesive liquid embolic agents (e.g.Onyx (ev3, Irvine, CA), glue, gelatin sponge or thrombin, if the persisting collaterals could not be catheterised,.

## Conclusion

Endovascular repair of de novo post stenotic aortic coarctation is a safe and durable technique that provides a less invasive alternative to open surgical repair. An endovascular approach allows exclusion of complex collateralised aneurysms without the additional haemorrhage risk of open surgical repair. The jailed sheath technique allows for complete selective embolisation to ensure technical success without a type II endoleak.

## Data Availability

Not Applicable.
